# Healthy Japanese dietary pattern is associated with slower biological aging in older men: WASEDA’S health study

**DOI:** 10.3389/fnut.2024.1373806

**Published:** 2024-05-24

**Authors:** Takuji Kawamura, Mitsuru Higuchi, Tomoko Ito, Ryoko Kawakami, Chiyoko Usui, Kristen M. McGreevy, Steve Horvath, Radak Zsolt, Suguru Torii, Katsuhiko Suzuki, Kaori Ishii, Shizuo Sakamoto, Koichiro Oka, Isao Muraoka, Kumpei Tanisawa

**Affiliations:** ^1^Waseda Institute for Sport Sciences, Waseda University, Saitama, Japan; ^2^Research Center for Molecular Exercise Science, Hungarian University of Sports Science, Budapest, Hungary; ^3^Faculty of Sport Sciences, Waseda University, Saitama, Japan; ^4^Department of Food and Nutrition, Tokyo Kasei University, Tokyo, Japan; ^5^Physical Fitness Research Institute, Meiji Yasuda Life Foundation of Health and Welfare, Tokyo, Japan; ^6^Center for Liberal Education and Learning, Sophia University, Tokyo, Japan; ^7^Department of Biostatistics, Fielding School of Public Health, University of California, Los Angeles, Los Angeles, CA, United States; ^8^Department of Human Genetics, David Geffen School of Medicine, University of California, Los Angeles, Los Angeles, CA, United States; ^9^Altos Labs, San Diego Institute of Science, San Diego, CA, United States; ^10^Faculty of Sport Science, Surugadai University, Saitama, Japan

**Keywords:** aging, biological clocks, dietary patterns, DNA methylation, epigenetics, Japanese

## Abstract

Aging is the greatest risk factor for numerous diseases and mortality, and establishing geroprotective interventions targeting aging is required. Previous studies have suggested that healthy dietary patterns, such as the Mediterranean diet, are associated with delayed biological aging; however, these associations depend on nationality and sex. Therefore, this study aimed to investigate the relationship between dietary patterns identified through principal component analysis and biological aging in older men of Japan, one of the countries with the longest life expectancies. Principal component analysis identified two dietary patterns: a healthy Japanese dietary pattern and a Western-style dietary pattern. Eight epigenetic clocks, some of the most accurate aging biomarkers, were identified using DNA methylation data from whole-blood samples. Correlation analyses revealed that healthy Japanese dietary patterns were significantly negatively or positively correlated with multiple epigenetic age accelerations (AgeAccel), including AgeAccelGrim, FitAgeAccel, and age-adjusted DNAm-based telomere length (DNAmTLAdjAge). Conversely, the Western-style dietary pattern was observed not to correlate significantly with any of the examined AgeAccels or age-adjusted values. After adjusting for covariates, the healthy Japanese dietary pattern remained significantly positively correlated with DNAmTLAdjAge. Regression analysis showed that healthy Japanese dietary pattern contributed less to epigenetic age acceleration than smoking status. These findings suggest that a Western-style dietary pattern may not be associated with biological aging, whereas a healthy Japanese dietary pattern is associated with delayed biological aging in older Japanese men. Our findings provide evidence that healthy dietary patterns may have mild beneficial effects on delayed biological aging in older Japanese men.

## 1 Introduction

Daily nutritional intake and diet are essential requirements for a healthy life, and overeating and malnutrition are closely associated with the development of metabolic, cardiovascular, and neurodegenerative diseases. Nutrient intake and diet are closely associated with aging and have been implicated in lowering the risk of age-related diseases and mortality (1). In recent years, an approach based on the “geroscience hypothesis” has been expanding, establishing intervention strategies that target the greatest risk factor for these diseases, aging itself, rather than addressing individual diseases (2, 3). From an economic perspective, it has been shown that a reduction in morbidity that improves health is more valuable than further increases in life expectancy and that targeting aging offers potentially larger economic gains than eradicating individual diseases (4). Given this background, research is underway to evaluate the efficacy of geroprotectants in delaying aging (5). However, evidence for the geroprotective effects of nutritional intake and diet is lacking.

Aging is a complex, multifaceted phenomenon influenced by genetic, environmental, and lifestyle factors and is characterized by a gradual decline in the physiological function and resilience of body systems over time. Epigenetic alterations have received increasing attention in recent years as hallmarks of aging, and epigenetic clocks based on age-related changes in DNA methylation patterns are promising biomarkers for testing the efficacy of geroprotective interventions (6). Most epigenetic clocks are calculated by (1) selecting key cytosine-phosphate-guanine (CpG) sites where hyper- and hypomethylation correlate with age and other phenotypes, weighting them with a linear model, and (2) creating an equation to estimate age based on the methylation level of each CpG site (7). Epigenetic clocks are associated with chronological age, onset of age-related diseases, and mortality (8, 9) and are considered highly accurate biomarkers of an individual’s biological aging. Therefore, the epigenetic clock may be a useful biomarker for assessing the geroprotective effects of nutritional intake and diet.

Accumulating evidence suggests that the epigenetic clock is associated with lifestyle factors, such as body composition (10), physical activity (11, 12) and physical fitness levels (13, 14), smoking (15), and alcohol intake (15), and can be delayed or reversed by pharmacological and lifestyle interventions (16–19). It has also been suggested that healthy dietary patterns, diets rich in fruits and vegetables, and mediterranean diets may delay the epigenetic clocks (20–25). However, these studies have primarily focused on Western populations, and there is no literature available on Asians, who have a very different dietary culture, especially the Japanese, one of the longest-lived countries in the world. Considering that the Mediterranean diet rejuvenated the epigenetic clock in a nationality- and sex-specific manner in a limited nutritional intervention study (26), the relationship between dietary patterns and the epigenetic clock is expected to vary greatly depending on the characteristics of the study participants. We found that a rich intake of micronutrients, such as vitamins and minerals, was associated with delayed epigenetic clocks in older Japanese men, even after adjusting for body mass index (BMI) and smoking (14). However, when recommending geroprotective intervention strategies, it is necessary to investigate the relationship with the epigenetic clock, not on the basis of nutrient levels, but on a more comprehensive unit of dietary patterns. In this study, given the potential sex-based differences in terms of epigenetic age and age acceleration, as well as the limited sample size of older women, we decided to conduct our study only in the older men from the study cohort.

The purpose of this study was to investigate the relationship between dietary patterns identified via principal component analysis (PCA) and epigenetic clocks. Specifically, we conducted a dietary survey in older men aged 65–72 years and identified two characteristic dietary patterns using PCA: a healthy Japanese dietary pattern and a Western-style dietary pattern. We also calculated eight epigenetic clocks: Horvath clock, Hannum clock, BioAge4HAStatic, DNAmSkinBloodClock, DNAmPhenoAge, DNAmGrimAge, DNAmFitAge, and DNAm-based telomere length (DNAmTL) using the methylation data of DNA samples extracted from the whole blood of the participants. Through these surveys and measurements, we identified dietary patterns associated with delayed biological aging.

## 2 Materials and methods

### 2.1 Participants

The study was conducted using the same procedures as in our previous study on participants of the Waseda Alumni’s Sports, Exercise, Daily Activity, Sedentariness, and Health Study (WASEDA’S Health Study) (14, 27–32). Briefly, this study included 169 men aged 65–72 years who participated in the baseline survey of Cohort D between March 2015 and March 2020 [see our previous study for details of the cohort (30)], 144 of whom were included in the order of measurement date from the earliest to the latest, after excluding those whose DNA sample quality did not meet the criteria (*n* = 11). The participants were briefed about the study and signed an informed consent form prior to the baseline survey. This study was approved by the Research Ethics Committee of Waseda University (approval numbers: 2014-G002 and 2018-G001) and conducted in accordance with the Declaration of Helsinki (1964).

### 2.2 Self- administered questionnaires and dietary assessment

Age (in years), smoking habits (current, former, and non-smoker), frequency of alcohol consumption (less than once a week, 2–4 times a week, and ≥ 5 times a week), exercise habit (yes and no), and socioeconomic factors, such as marital status (married and single), education status (high school, junior college and technical college, and college diploma), and income level (< 3,000,000 JPY, 3,000,000 JPY–5,000,000 JPY, 5,000,000 JPY–7,000,000 JPY, 7,000,000 JPY–10,000,000 JPY, and ≥ 10,000,000 JPY) were investigated using a self-administered questionnaire. Dietary intake status was assessed using a brief self-administered dietary history questionnaire (BDHQ) using the same methods as in previous studies (27, 28, 31, 32). Briefly, the BDHQ consists of four pages and takes approximately 15 min to complete. After answering the questions, the examiner carefully checked to ensure there were no erroneous answers. Based on the Japanese Standard Tables of Food Composition (33), ad hoc computer algorithms for the BDHQ were used to estimate the dietary intakes of 58 food and beverage items, energy, and selected nutrients. The validity of the dietary intake data (energy, nutrients, and foods) assessed using the BDHQ was confirmed using 16-d semi-weighted dietary records as the gold standard (34, 35).

### 2.3 Anthropometric measurement

Height (cm) was measured using a stadiometer (YHS-200D; YAGAMI Inc., Nagoya, Japan). Body weight (kg) and fat content (%) were measured using a multifrequency bioelectrical impedance analyzer (MC-980A; Tanita, Tokyo, Japan) with light clothing and without shoes. BMI (kg/m^2^) was calculated using height and body weight measurements.

### 2.4 Blood sampling, DNA extraction, and measurement of epigenome-wide DNA methylation

The procedure from blood collection to sample storage has been described previously (14, 30). The participants were instructed to fast for at least 12 h the night before blood collection. Venous blood was collected from the forearm vein in a collection tube containing an anticoagulant (EDTA-2Na). DNA was extracted from whole blood using a QIAamp DNA Midi Kit (Qiagen, Germany) according to the manufacturer’s instructions. Extracted DNA was dissolved in Buffer AE (10 mM Tris-Cl, 0.5 mM EDTA, pH 9.0). Prior to DNA methylation measurements, DNA samples were adjusted to a concentration of ≥50 ng/μL and A260/280 purity in the range of 1.7–2.1. Epigenome-wide DNA methylation was measured using the same procedure as previously reported (12). Briefly, bisulfite conversion of genomic DNA was performed using the EZ DNA Methylation Kit (Zymo Research, Irvine, CA, USA), followed by hybridization using the Infinium MethylationEPIC BeadChip Kit (Illumina Inc., San Diego, CA, USA). Sample- and probe-based quality checks were performed using the minfi, meffil, and ewastools R packages (13). In this study, all 144 DNA samples met the criteria set by Illumina, and no samples were excluded.

### 2.5 Calculation of epigenetic clocks

Eight epigenetic clocks—the Horvath clock, Hannum clock, BioAge4HAStatic, DNAmSkinBloodClock, DNAmPhenoAge, DNAmGrimAge, DNAmFitAge, and DNAmTL—and age acceleration or age-adjusted values for each of these, were calculated using Horvath’s online age calculator^1^ and the epigenome-wide DNA methylation data (21, 22, 36–41). The Horvath clock is a “pan-tissue clock” that was developed using samples containing 51 healthy tissues and cell types, including blood, brain, and skeletal muscle, and utilizes 353 CpG sites to predict chronological age of multiple tissues (36). Since the Horvath clock does not work reliably in cultured cells, especially fibroblasts, the DNAmSkinBloodClock was developed to better predict chronological age of human fibroblasts, keratinocytes, buccal cells, endothelial cells, lymphoblastoid cells, skin, blood and saliva samples (37). While these multi-tissue age predictors have been developed, the Hannum Clock is a blood-based tissue-specific age predictor that predicts chronological age from 71 CpG sites (38). The BioAge4HAStatic clock was built upon the Hannum clock and defined by forming a weighted average of Hannum’s estimate with 3 cell types that exhibit age-related changes (39). DNAmPhenoAge (21), DNAmGrimAge (22), DNAmFitAge (40), and DNAmTL (41) were developed as predictors of morbidity and mortality and are also referred to as composite epigenetic clocks. The DNAmPhenoAge is a biological age estimator calculated from 513 CpG sites that combines chronological age with nine other biomarkers, including albumin, creatinine, glucose, C-reactive protein levels, lymphocyte percentage, mean cell volume, red blood cell distribution width, alkaline phosphatase, and white blood cell count (21). The DNAmGrimAge is also a biological estimator calculated from 1030 CpG sites that combines with seven DNA methylation-based plasma protein markers, including cystatin C, leptin, tissue metalloproteinase 1 inhibitor, adrenomedullin, β2-microglobulin, growth differentiation factor 15, and plasminogen activator inhibitor 1, and smoking pack-years, which is shown to be associated with morbidity or mortality (22). The DNAmFitAge is a newly developed biological age estimator that incorporates the DNAmGrimAge with blood-based DNAm biomarkers of fitness parameters such as walking speed, maximum grip strength, forced expiratory volume in one second, and maximal oxygen uptake (40). DNAmTL is a DNAm-based biomarker calculated from 140 CpG sites that accurately predicts leukocyte telomere length (LTL) (41). DNAmTL not only accurately predicts LTL, but also shows stronger predictive power for morbidity and mortality compared to LTL. These four composite epigenetic clocks can predict biological status and age with higher accuracy than the four epigenetic clocks trained on chronological age alone (7).

### 2.6 Covariates

Both epigenetic age acceleration and age-adjusted values for the statistical analyses in this study were adjusted for chronological age. Referring to a previous related study (25), BMI, smoking and drinking statuses, exercise habits, and socioeconomic factors, such as marital status, education status, and income level, were selected as covariates and included in the statistical analyses presented in the following subsection. The methods used to calculate BMI and survey smoking and drinking statuses, exercise habits, as well as socioeconomic factors, such as marital status, education status, and income level are described above.

### 2.7 Statistical analyses

To identify dietary patterns, we performed PCA based on energy-adjusted food intake using the density method for 52 food and beverage items, as previously described (27). More specifically, of the 58 items, six items (sugar added to coffee and black tea, three items usually added during cooking [salt, oil, and sugar], table salt and salt-containing seasoning at the table, and soup consumed with noodles) were excluded from the analysis (27). To determine the number of factors to retain, we considered conventional methods such as eigenvalues, slope of scree plots, and factor interpretability (42). Of the 18 factors with eigenvalues of > 1, the scree plot decreased significantly after the second (from 5.80 to 3.57) and fourth factors (from 3.14 to 2.58). Given these, we narrowed it down to the third factor and, for interpretability reasons, ultimately excluded it. In the end, two factors were retained as a result of the PCA. The dietary patterns were named according to the food items that showed high loading (absolute value) based on the two included factors. The factor scores for each dietary pattern and for each individual were calculated by summing the intakes of the food items weighted according to their factor loadings. As descriptive data, continuous variables are presented as mean ± standard deviation (SD) and categorical variables are presented as number of persons and percentages. The distributions of the two dietary pattern scores were confirmed using histogram plots, and as normality was not assumed, Spearman’s rank correlation analyses were performed to determine the correlation between each epigenetic age acceleration and the two identified dietary patterns. In parallel, because the two dietary pattern scores contained negative values, they were log-transformed after adding +5, followed by Pearson correlation and partial correlation analyses. A partial correlation analysis adjusted for BMI, smoking and drinking status, exercise habit, and socioeconomic factors, such as marital status, education status, and income level, was performed. We performed a post-hoc power analysis and used the same methods described in a previous study (14). Assuming a sample size of 144 and an *r* value of 0.235, the statistical power of Spearman’s correlation analysis was 0.800; assuming a sample size of 144 and an ρ value of 0.231, the statistical power of Pearson’s correlation analysis was 0.800. The statistical power of the partial correlation analysis was 0.800, assuming a sample size of 140 and *r* value of 0.236. In addition to these correlation analyses, we performed single and multiple regression analyses. Single regression analysis was performed with each epigenetic age acceleration and age-adjusted value as dependent variables and log-transformed dietary pattern scores as independent variables, and regression coefficients (B), 95% confidence intervals (95% CI), and coefficient of determination (*R*^2^) were calculated. Multiple regression analysis was performed with each epigenetic age acceleration and age-adjusted value as dependent variables and log-transformed dietary pattern scores, BMI, smoking and drinking status, exercise habits, and socioeconomic factors as independent variables. Partial regression coefficients (B), 95% CI, and standard partial regression coefficients (β) were calculated. Forced entry methods were used to develop the model. Before using the forced entry method, variance inflation factors (VIF) were calculated for each variable to avoid multicollinearity and to ensure that the VIF was less than 10. All the statistical analyses were performed using SPSS version 26 (IBM Corporation, Chicago, IL, USA).

## 3 Results

### 3.1 Dietary patterns

**FIGURE 1:**
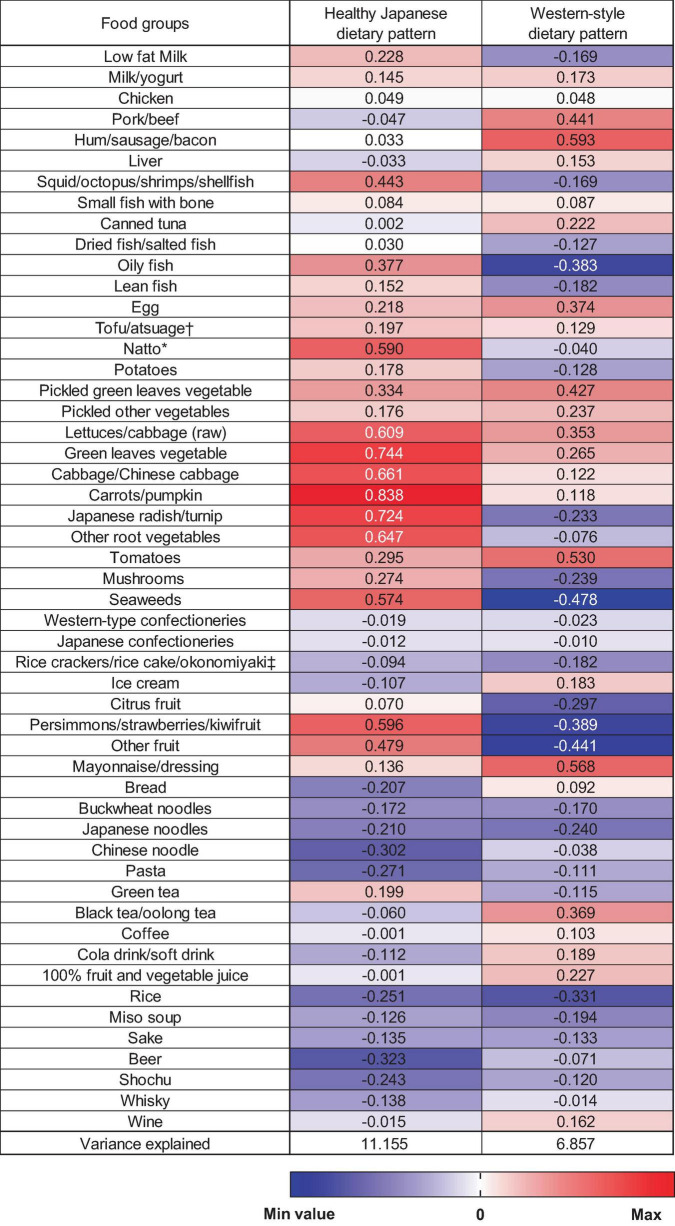
Factor loading matrix for dietary patterns identified by the principal component analysis. *Fermented soybeans. ^†^Deep-fried tofu. ^‡^Savoury pancake with various ingredients (meat, fish, and vegetable).

In this study, two dietary patterns were identified using PCA (Figure 1). The first factor was the “healthy Japanese dietary pattern,” which is characterized by a high intake of vegetables, fruits, soy products, and seafood. The second factor was named the “Western-style dietary pattern” based on the characteristics of a high intake of meat and processed meats, eggs, and mayonnaise dressings. These two dietary patterns accounted for 11.2% and 6.9% of the variance in food intake and explained 18.0% as cumulative. Although this cumulative contribution percentage does not adequately summarize the information on the overall dietary pattern, we were able to identify two characteristic dietary patterns—including the healthy Japanese dietary pattern (27, 28) —which we aimed to examine in terms of their associations with epigenetic clocks.

### 3.2 Characteristics of the participants

The predicted age values for each epigenetic clock were 62.2 ± 4.5 years for the Horvath clock, 55.1 ± 3.8 years for the Hannum clock, 52.3 ± 4.7 years for the BioAge4HAStatic, 65.7 ± 3.0 years for the DNAmSkinBloodClock, 56.7 ± 5.5 years for the DNAmPhenoAge, 69.2 ± 3.4 years for the DNAmGrimAge, 71.7 ± 3.5 years DNAmFitAge, and 6.6 ± 0.2 for DNAmTL, respectively. Age acceleration and age-adjusted values for each epigenetic clock, and other variables used in this study are listed in Table 1. The results of the correlation analysis of age, epigenetic age, epigenetic age acceleration, or age-adjusted values are presented in Supplementary Tables 1, 2.

**TABLE 1:** Characteristics of the participants (*n* = 144).

	Mean ± SD
Age (years)	68 ± 1.9
Height (cm)	168 ± 5.8
Body weight (kg)	66.1 ± 8.2
BMI (kg/m^2^)	23.4 ± 2.5
Body fat (%)	21.2 ± 5.4
**Dietary pattern scores**	
Healthy Japanese dietary pattern	0 ± 1.0 [−1.5 to 7.2]
Western-style dietary pattern	0 ± 1.0 [−3.5 to 5.6]
Horvath clock (predicted years)	62.2 ± 4.5
Hannum clock (predicted years)	55.1 ± 3.8
BioAge4HAStatic (predicted years)	52.3 ± 4.7
DNAmSkinBloodClock (predicted years)	65.7 ± 3
DNAmPhenoAge (predicted years)	56.7 ± 5.5
DNAmGrimAge (predicted years)	69.2 ± 3.4
DNAmFitAge (predicted years)	71.7 ± 3.5
DNAmTL (kb)	6.6 ± 0.2
AgeAccelHorvath	0 ± 4.3
AgeAccelHannum	0 ± 3.7
BioAge4HAStaticAdjAge	0 ± 4.6
DNAmSkinBloodClockAdjAge	0 ± 2.8
AgeAccelPheno	0 ± 5.5
AgeAccelGrim	0 ± 3.2
FitAgeAccel	0 ± 3.3
DNAmTLAdjAge	0 ± 0.2
**Smoking status**	
Non-smoker	44 (30.6%)
Past smoker	90 (62.5%)
Current smokers	10 (6.9%)
**Drinking status**	
0–1 times/week	41 (28.5%)
2–4 times/week	27 (18.7%)
5–7 times/week	76 (52.8%)
**Exercise habit**	
Yes	117 (81.3%)
No	27 (18.7%)
**Marital status**	
Married	135 (96.4%)
Single	5 (3.6%)
**Education status**	
High school	1 (0.7%)
Junior college and technical college	1 (0.7%)
College diploma	138 (98.6%)
**Income status**	
< 3,000,000 JPY	10 (7.2%)
3,000,000 JPY ≤ 5,000,000 JPY	50 (35.7%)
5,000,000 JPY ≤ 7,000,000 JPY	38 (27.1%)
7,000,000 JPY ≤ 10,000,000 JPY	19 (13.6%)
10,000,000 JPY ≤	23 (16.4%)

Data are mean ± SD (Dietary pattern scores also indicated the range). BMI, body mass index; AgeAccel, age acceleration; AdjAge, age adjusted values; TL, telomere length.

### 3.3 Correlations between dietary pattern score and epigenetic clocks

**FIGURE 2:**
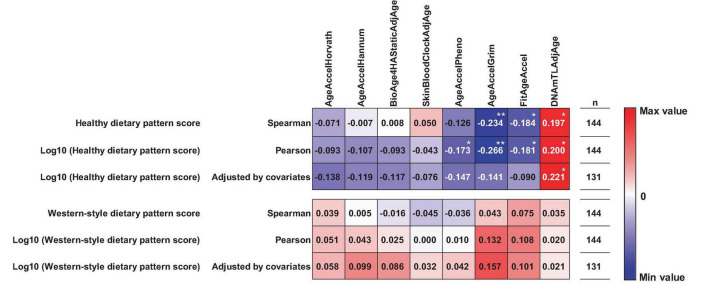
Correlations between dietary patterns and epigenetic clocks. The values in the figure represent correlation and partial correlation coefficients. Partial correlation analysis was performed adjusted for body mass index (BMI), smoking and drinking status, exercise habit, and socioeconomic factors, such as marital status, education status, and income level. AgeAccel, age acceleration; AdjAge, age adjusted values; TL, telomere length. Significant correlations at *p* < 0.05 and *p* < 0.01 are indicated by * and **.

Figure 2 shows the results of correlation analysis between the two dietary pattern scores and each epigenetic clock. For the healthy Japanese dietary pattern, both the actual and log-transformed values were significantly correlated with the composite epigenetic clocks. More specifically, healthy Japanese dietary pattern scores had significant negative correlations with AgeAccelGrim (ρ = −0.234; *p* = 0.005) and FitAgeAccel (ρ = −0.184; *p* = 0.027) and significant positive correlations with DNAmTLAdjAge (ρ = 0.197; *p* = 0.018) (correlation scatterplots are shown in Supplementary Figure 1). The log-transformed healthy Japanese dietary pattern scores also had significant negative correlations with AgeAccelPheno (*r* = −0.173; *p* = 0.038), AgeAccelGrim (*r* = −0.266; *p* = 0.013), and FitAgeAccel (*r* = −0.181; *p* = 0.030) and significant positive correlations with DNAmTLAdjAge (*r* = 0.200; *p* = 0.016) (correlation scatterplots are shown in Supplementary Figure 2). There were significant positive correlations between the log-transformed healthy Japanese dietary pattern scores and DNAmTLAdjAge (*r* = 0.221; *p* = 0.036), even after adjusting for BMI, smoking and drinking status, exercise habit, and, and socioeconomic factors. In contrast, there were no significant correlations between healthy Japanese dietary pattern scores and the AgeAccelHorvath, AgeAccelHannum, BioAge4HAStaticAdjAge, DNAmSkinBloodClockAdjAge, Age AccelPheno, AgeAccelGrim, and FitAgeAccel. For the Western-style dietary pattern, neither the actual nor the log-transformed values could be observed to correlate significantly with any of the epigenetic clocks in any correlation analyses.

### 3.4 Single and multiple regression analysis

The results of single regression analysis between the two dietary pattern scores and each epigenetic clock are shown in Supplementary Table 3. For the healthy Japanese dietary pattern, significant regression models were obtained for AgeAccelPheno (*B* = −10.474; *p* = 0.035), AgeAccelGrim (*B* = −9.152; *p* = 0.001), FitAgeAccel (*B* = −6.488; *p* = 0.029), and DNAmTLAdjAge (*B* = 0.372; *p* = 0.016). However, no significant regression models were obtained for the other four epigenetic clocks. For the Western-style dietary pattern, the regression models were not significant for any of the clocks.

The results of multiple regression analysis of each epigenetic clock are shown in Supplementary Tables 4, 5. For the healthy Japanese dietary pattern, significant regression models were obtained only for AgeAccelGrim (Model *R*^2^ = 0.167; *p* < 0.001) and FitAgeAccel (Model *R*^2^ = 0.066; *p* = 0.029). However, no significant regression models were obtained for the other epigenetic clocks. For the Western-style dietary pattern, significant regression models were obtained only for AgeAccelGrim (Model *R*^2^ = 0.171; *p* = 0.029) and FitAgeAccel (Model *R*^2^ = 0.068; *p* = 0.026). However, no significant regression models were obtained for the other epigenetic clocks. In the above four multiple regression models, the standard partial regression coefficient (β) was consistently highest in smoking status.

## 4 Discussion

This study revealed that a healthy Japanese dietary pattern exhibited significant negative or positive correlations with AgeAccelGrim, FitAgeAccel, and DNAmTLAdjAge in older Japanese men. Conversely, the Western-style dietary pattern was not observed to correlate significantly with any of the examined epigenetic clocks. After adjusting for covariates, the healthy Japanese dietary pattern remained significantly positively correlated with DNAmTLAdjAge. Regression analysis showed that healthy Japanese dietary pattern contributed less to epigenetic age acceleration than smoking status. These findings suggest that a Western-style dietary pattern is possibly not associated with biological aging, whereas a healthy Japanese dietary pattern is mildly associated with delayed biological aging in older Japanese men.

Several previous studies have examined the relationship between dietary habits and the epigenetic clock based on individual dietary components, such as food items and nutrients. Collectively, the findings of these studies suggest a negative correlation between the intake of poultry, fish, vegetables, and fruit products and age acceleration and a positive correlation between red meat intake and age acceleration (20–22). In addition, studies have consistently shown that antioxidants in the blood are negatively correlated with accelerated age (20–22). Prior to the present study, we also found a negative correlation between estimated daily intake of vitamin C, β-carotene, iron, and copper and epigenetic age acceleration (14). However, previous research approaches lack a comprehensive understanding of the beneficial effects of daily dietary habits and lack specificity in establishing dietary guidelines and recommending dietary habit improvements.

To address this issue, Kresovich et al. (23) calculated scores for four dietary patterns [Dietary Approaches to Stop Hypertension diet, Healthy Eating Index-2015, Alternative Healthy Eating Index (aHEI-2010), and Alternative Mediterranean diet] based on the Block Food Frequency Questionnaire (Block FFQ) in 2694 women with an average age of 56 years and found that the scores for all four dietary patterns were negatively correlated with epigenetic age acceleration, most notably with PhenoAgeAccel and GrimAgeAccel. Of the four dietary patterns in this study, aHEL2010 had the strongest association with epigenetic age acceleration; similar results were reported in subsequent studies (15). Kim et al. (24) also evaluated the Dietary Approaches to Stop Hypertension (DASH) score in 1995 using men and women with an average age of 67 years and found that higher scores were correlated with lower epigenetic age accelerations [i.e., Dunedin PoAm (43), PhenoAgeAccel, and GrimAgeAccel]. Thomas et al. (25) reported a negative correlation between adherence to a Mediterranean diet and PhenoAgeAccel. Taken together, dietary patterns, such as aHEl-2010, DASH, and the Mediterranean diet, which are associated with a variety of diseases and mortality, appear to be associated with composite epigenetic clocks that can capture biological age.

Unlike previous studies based on dietary patterns, the present study identified two dietary patterns using PCA based on the results of the dietary assessment. These were the healthy Japanese dietary pattern and the Western-style dietary pattern, of which the healthy Japanese dietary pattern score was associated with epigenetic age acceleration and age-adjusted value of the composite epigenetic clocks that developed as predictors of morbidity and mortality in older Japanese men. The healthy Japanese dietary pattern is characterized by vegetables, fruits, seaweed, and natto (fermented soybeans) and is aligned to some extent with the dietary patterns used in previous studies, such as the aHEl-2010, DASH, and Mediterranean diets. Our findings provide two key insights: (1) that a healthy Japanese dietary pattern delays the biological aging processes; and (2) that the Western dietary pattern may not have adversely effect on this process in older Japanese men. Our findings also suggest that composite epigenetic clocks, particularly DNAmTLAdjAge, may represent a target for predicting the impact of dietary patterns on future morbidity and mortality, providing insights into the relationship between diet and the aging process, which remains poorly understood. However, it should be mentioned that based on the results of the multiple regression analysis, the contribution of smoking to the epigenetic clocks were higher than that of healthy Japanese dietary pattern and Western style dietary pattern. This result is consistent with our previous report (14), in which multiple regression analysis was performed including more lifestyle factors, highlighting the negative influence of smoking on biological aging.

The findings of the present study, based on the two categories of healthy Japanese- and Western-style dietary patterns, may provide important insights into establishing ideal dietary patterns for older Japanese men. However, the present study has several limitations. First, this study was based on a cross-sectional design, and the causal relationship between dietary patterns and the epigenetic clocks are unknown. Limited evidence from intervention studies suggests that combined lifestyle interventions, including dietary and exercise habits, decrease epigenetic age acceleration as compared to the control group (17, 18). Therefore, future longitudinal studies should follow both healthy Japanese dietary pattern scores and epigenetic age acceleration to clarify the causal relationship between these two factors. Second, this study included only older Japanese men, which limits the generalizability of the present findings. Several interventional studies have demonstrated that the effects of delayed epigenetic clock progression due to supplementation and altered dietary habits differ according to genotype, nationality, and sex (26, 44). Further studies are needed to elucidate whether the findings of this study can be replicated in different populations, such as women and different nationalities. Third, we used self-reported data by BDHQ to identify the dietary patterns; however, we cannot exclude the possibility that recall bias may have occurred. Fourth, the small sample size of this study may have resulted in insufficient statistical power and type II errors. Addressing these limitations would strengthen the validity and generalizability of the study findings and provide a more comprehensive understanding of the relationship between dietary patterns and biological aging.

## 5 Conclusion

The Western-style dietary pattern was observed not to be associated with epigenetic age acceleration and age-adjusted values, whereas the healthy Japanese dietary pattern was associated with multiple composite epigenetic age accelerations and age-adjusted value that developed as predictors of morbidity and mortality in older Japanese men, suggesting that a healthy Japanese dietary pattern may have mildly beneficial effects on delayed biological aging in older Japanese men.

## Data availability statement

The datasets presented in this article are not readily available because this study is ongoing. However, the measurement data used to support the results of this study are available from the corresponding author upon reasonable request. Requests to access the datasets should be directed to TK, kawamura.takuji@tf.hu.

## Ethics statement

The studies involving humans were approved by the Research Ethics Committee of Waseda University. The studies were conducted in accordance with the local legislation and institutional requirements. The participants provided their written informed consent to participate in this study.

## Author contributions

TK: Conceptualization, Data curation, Formal analysis, Funding acquisition, Investigation, Methodology, Writing – original draft. MH: Conceptualization, Investigation, Methodology, Project administration, Supervision, Writing – review and editing. TI: Investigation, Methodology, Writing – review and editing. RK: Investigation, Methodology, Writing – review and editing. CU: Investigation, Methodology, Writing – review and editing. KM: Formal analysis, Methodology, Writing – review and editing. SH: Formal analysis, Methodology, Writing – review and editing. RZ: Methodology, Writing – review and editing. ST: Writing – review and editing, Investigation, Methodology. KS: Investigation, Methodology, Writing – review and editing. KI: Project administration, Writing – review and editing. SS: Investigation, Methodology, Writing – review and editing, Supervision. KO: Supervision, Writing – review and editing. IM: Supervision, Writing – review and editing. KT: Conceptualization, Investigation, Methodology, Supervision, Writing – review and editing.

## Footnotes

1
https://dnamage.genetics.ucla.edu/
