# Supplementary material for: Healthy Japanese dietary pattern is associated with slower biological aging in older men: WASEDA’S health study
Source: Front Nutr. 2024 May 24;11:1373806. doi: 10.3389/fnut.2024.1373806 (PMC11157009; doi:10.3389/fnut.2024.1373806)
Supplement: Supplementary file 1 [file Table_1.pdf]

Supplemental data

Table S1. Correlations between chronological age and each epigenetic age (predicted age).

		Horvath Clock	Hannum Clock	BioAge4HAStatic	DNAmAge SkinBloodClock	DNAmPhenoAge	DNAmGrimAge	DNAmFitAge	DNAmTL
Chronological Age	*r*	0.083	**0.232****	**0.217****	**0.317****	0.057	**0.379****	**0.368****	-0.139
	p	0.321	**0.005**	**0.009**	**< 0.001**	0.501	**< 0.001**	**< 0.001**	0.097
	n	144	**144**	**144**	**144**	144	**144**	**144**	144

TL, telomere length. Significant correlations at $p < 0.01$ are indicated by **.

Table S2. Correlations between each epigenetic age acceleration and age-adjusted values.

		AgeAccelHorvath	AgeAccelHannum	BioAge4HAStatic AdjAge	SkinBloodClock AdjAge	AgeAccelPheno	AgeAccelGrim	FitAgeAccel	DNAmTL AdjAge
AgeAccelHorvath	*r*		**0.527****	**0.510****	**0.469****	**0.356****	0.135	**0.395****	**-0.212***
	p		**< 0.001**	**< 0.001**	**< 0.001**	**< 0.001**	0.107	**< 0.001**	**0.011**
	n		**144**	**144**	**144**	**144**	144	**144**	**144**
AgeAccelHannum	*r*	**0.527****		**0.962****	**0.561****	**0.502****	**0.207***	**0.476****	**-0.305****
	p	**< 0.001**		**< 0.001**	**< 0.001**	**< 0.001**	**0.013**	**< 0.001**	**< 0.001**
	n	**144**		**144**	**144**	**144**	**144**	**144**	**144**
BioAge4HAStatic AdjAge	*r*	**0.510****	**0.962****		**0.538****	**0.526****	**0.211***	**0.492****	**-0.336****
	p	**< 0.001**	**< 0.001**		**< 0.001**	**< 0.001**	**0.011**	**< 0.001**	**< 0.001**
	n	**144**	**144**		**144**	**144**	**144**	**144**	**144**
SkinBloodClock AdjAge	*r*	**0.469****	**0.561****	**0.538****		**0.302****	0.126	**0.391****	**-0.181***
	p	**< 0.001**	**< 0.001**	**< 0.001**		**< 0.001**	0.132	**< 0.001**	**0.030**
	n	**144**	**144**	**144**		**144**	144	**144**	**144**
AgeAccelPheno	*r*	**0.356****	**0.502****	**0.526****	**0.302****		**0.455****	**0.546****	**-0.249****
	p	**< 0.001**	**< 0.001**	**< 0.001**	**< 0.001**		**< 0.001**	**< 0.001**	**0.003**
	n	**144**	**144**	**144**	**144**		**144**	**144**	**144**
AgeAccelGrim	*r*	0.135	**0.207***	**0.211***	0.126	**0.455****		**0.744****	**-0.208***
	p	0.107	**0.013**	**0.011**	0.132	**< 0.001**		**< 0.001**	**0.012**
	n	144	**144**	**144**	144	**144**		**144**	**144**
FitAgeAccel	*r*	**0.395****	**0.476****	**0.492****	**0.391****	**0.546****	**0.744****		**-0.491****
	p	**< 0.001**	**< 0.001**	**< 0.001**	**< 0.001**	**< 0.001**	**< 0.001**		**< 0.001**
	n	**144**	**144**	**144**	**144**	**144**	**144**		**144**
DNAmTL AdjAge	*r*	**-0.212***	**-0.305****	**-0.336****	**-0.181***	**-0.249****	**-0.208***	**-0.491****	
	p	**0.011**	**< 0.001**	**< 0.001**	**0.030**	**0.003**	**0.012**	**< 0.001**	
	n	**144**	**144**	**144**	**144**	**144**	**144**	**144**	

AgeAccel, age acceleration; AdjAge, age adjusted values; TL, telomere length. Significant correlations at $p < 0.05$ and $p < 0.01$ are indicated by * and **.

Table S3. Association between dietary patterns and epigenetic age acceleration and age-adjusted values by single regression analysis.

	B	95%CI			*p*	R^2
Healthy Japanese dietary pattern						
AgeAccelHorvath	-4.559	-12.527	–	3.409	0.260	0.009
AgeAccelHannum	-4.293	-10.846	–	2.26	0.197	0.012
BioAge4HAStaticAdjAge	-4.737	-12.924	–	3.451	0.255	0.009
DNAmSkinBloodClockAdjAge	-1.121	-6.184	–	3.942	0.662	0.001
AgeAccelPheno	-10.474	-20.179	–	-0.769	**0.035**	0.031
AgeAccelGrim	-9.152	-14.635	–	-3.667	**0.001**	0.071
FitAgeAccel	-6.488	-12.314	–	-0.662	**0.029**	0.033
DNAmTLAdjAge	0.372	0.071	–	0.673	**0.016**	0.040
Western-style dietary pattern						
AgeAccelHorvath	-0.105	-0.632	–	0.841	0.779	0.001
AgeAccelHannum	-0.062	-0.668	–	0.545	0.841	0.0003
BioAge4HAStaticAdjAge	-0.139	-0.895	–	0.618	0.717	0.001
DNAmSkinBloodClockAdjAge	-0.087	-0.553	–	0.379	0.714	0.001
AgeAccelPheno	-0.025	-0.932	–	0.882	0.957	0.00002
AgeAccelGrim	0.43	-0.089	–	0.949	0.104	0.019
FitAgeAccel	0.368	-0.174	–	0.910	0.181	0.013
DNAmTLAdjAge	0.008	-0.021	–	0.036	0.597	0.002

AgeAccel, age acceleration; AdjAge, age adjusted values; B: regression coefficient; CI: confidence interval; R^2: coefficient of determination: TL, telomere length.

Table S4. Multiple regression analysis of epigenetic age accelerations in the healthy Japanese dietary pattern.

	B	95%CI			*β*	VIF	*p*
AgeAccelGrim[*1]							
Intercept	3.066	-7.590	–	13.721			0.570
Log[10] (Healthy Japanese dietary pattern score)	-4.843	-10.723	–	1.038	-0.139	1.222	0.106
BMI	0.025	-0.180	–	0.230	0.020	1.094	0.809
Exercise habit	0.555	-0.722	–	1.833	0.069	1.064	0.391
Smoking status	-1.793	-2.688	–	-0.898	-0.326	1.127	**< 0.001**
Drinking status	-0.127	-0.307	–	0.053	-0.119	1.212	0.164
Marital status	1.639	-1.018	–	4.295	0.097	1.047	0.225
Education status	0.643	-2.002	–	3.287	0.038	1.062	0.631
Income status	-0.106	-0.526	–	0.314	-0.040	1.089	0.618
FitAgeAccel[*2]							
Intercept	5.403	-11.932	–	9.335			0.365
Log[10] (Healthy Japanese dietary pattern score)	-3.379	-0.322	–	7.650	-0.093	1.222	0.305
BMI	-0.169	-0.168	–	0.242	-0.127	1.094	0.140
Exercise habit	0.544	-0.806	–	1.738	0.064	1.064	0.447
Smoking status	-1.236	-0.332	–	0.004	-0.215	1.127	**0.015**
Drinking status	-0.128	-2.767	–	-0.991	-0.115	1.212	0.205
Marital status	1.202	-0.728	–	4.520	0.068	1.047	0.418
Education status	0.864	-2.249	–	3.012	0.049	1.062	0.559
Income status	-0.180	-0.522	–	0.315	-0.066	1.089	0.442

AgeAccel, age acceleration; B: partial regression coefficient; BMI: body mass index; CI: confidence interval; *β*: standard partial regression coefficient; VIF: variance inflation factor. [*1] $R^2 = 0.215$, adjusted $R^2 = 0.167$; [*2] $R^2 = 0.120$, adjusted $R^2 = 0.066$.

Table S5. Multiple regression analysis of epigenetic age accelerations in the western-style dietary pattern.

	B	95%CI			β	VIF	p
AgeAccelGrim *1							
Intercept	-1.298	-11.932	–	9.335			0.810
Log10 (Western-style dietary pattern score)	3.664	-0.322	–	7.650	0.142	1.027	0.071
BMI	0.037	-0.168	–	0.242	0.029	1.104	0.724
Exercise habit	0.466	-0.806	–	1.738	0.058	1.059	0.470
Smoking status	-1.879	-2.767	–	-0.991	-0.341	1.115	**< 0.001**
Drinking status	-0.164	-0.332	–	0.004	-0.154	1.064	0.056
Marital status	1.896	-0.728	–	4.520	0.112	1.027	0.155
Education status	0.382	-2.249	–	3.012	0.023	1.056	0.755
Income status	-0.104	-0.522	–	0.315	-0.039	1.088	0.625
FitAgeAccel *2							
Intercept	2.334	-9.412	–	14.079			0.695
Log10 (Western-style dietary pattern score)	2.594	-1.808	–	6.997	0.097	1.027	0.246
BMI	-0.161	-0.387	–	0.065	-0.121	1.104	0.162
Exercise habit	0.481	-0.924	–	1.886	0.057	1.059	0.499
Smoking status	-1.295	-2.276	–	-0.315	-0.226	1.115	**0.010**
Drinking status	-0.153	-0.339	–	0.032	-0.138	1.064	0.105
Marital status	1.381	-1.517	–	4.280	0.078	1.027	0.348
Education status	0.681	-2.224	–	3.586	0.039	1.056	0.644
Income status	-0.179	-0.641	–	0.284	-0.065	1.088	0.446

AgeAccel, age acceleration; B: partial regression coefficient; BMI: body mass index; CI: confidence interval; β: standard partial regression coefficient; VIF: variance inflation factor. *1 $R^2 = 0.219$, adjusted $R^2 = 0.171$; *2 $R^2 = 0.122$, adjusted $R^2 = 0.068$.

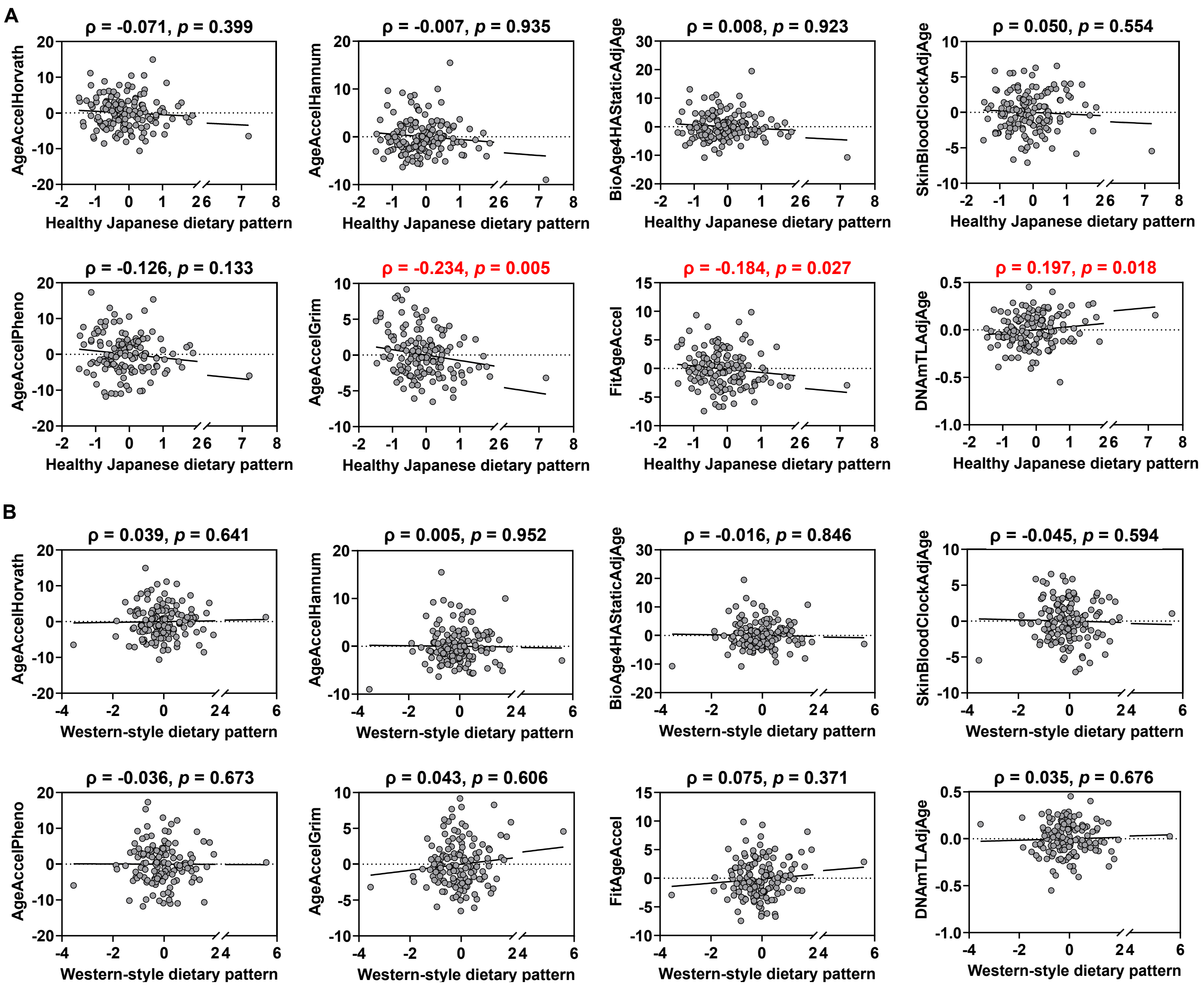

Figure S1. Spearman correlation scatterplots. **A)** Correlation between healthy Japanese dietary pattern scores and age acceleration (AgeAccel) and age adjusted (AdjAge) values for each epigenetic clock. **B)** Correlation between Western-style dietary pattern scores and AgeAccel and AdjAge. values for each epigenetic clock. Red letters indicate that the correlation is significant.

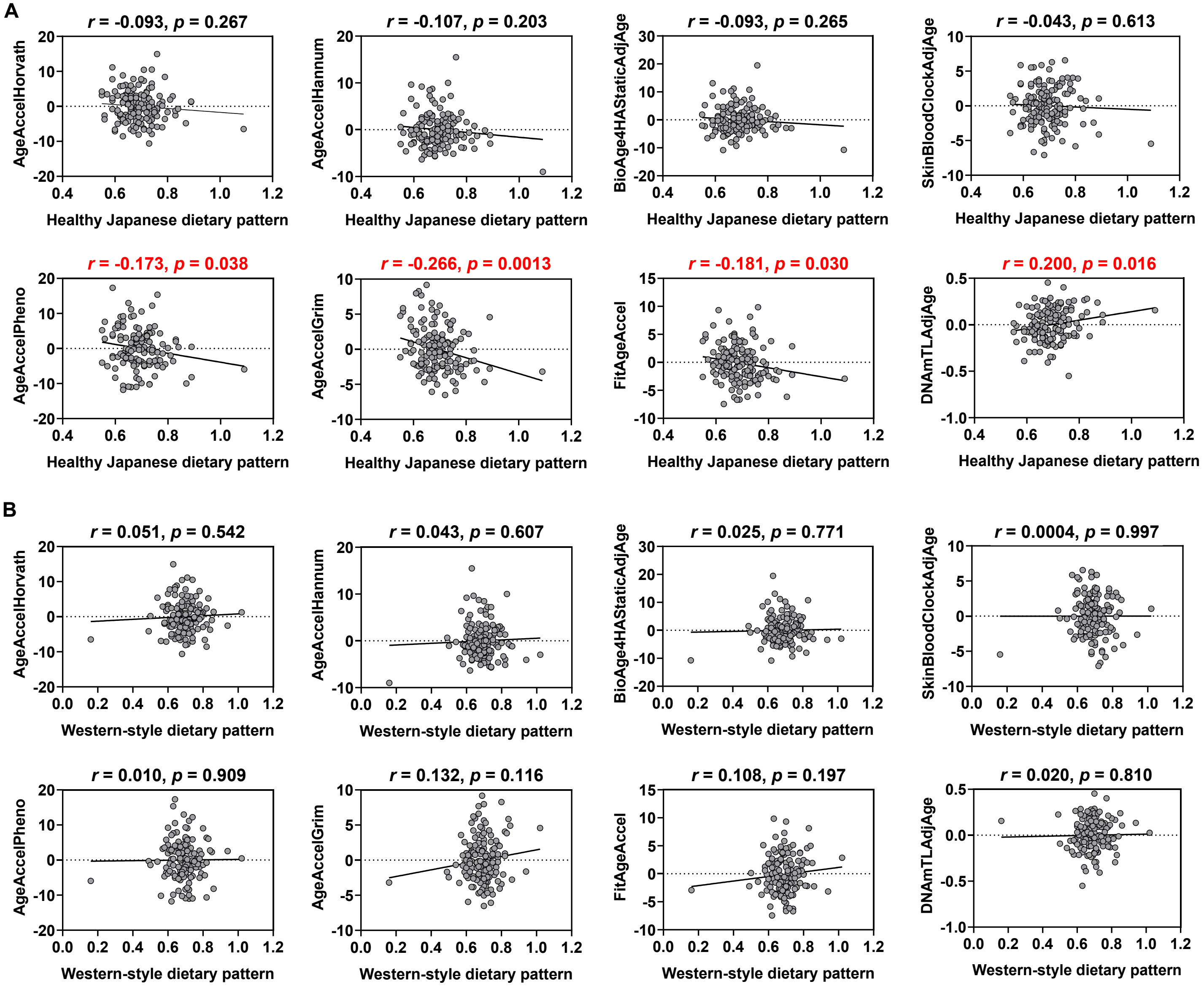

Figure S2. Pearson correlation scatterplots. **A)** Correlation between log10 (healthy Japanese dietary pattern scores) and age acceleration (AgeAccel) and age adjusted (AdjAge) values for each epigenetic clock. **B)** Correlation between log10 (western-style dietary pattern scores) and AgeAccel and AdjAge. values for each epigenetic clock. Red letters indicate that the correlation is significant.
